# Empowerment of personal injury victims through the internet: design of a randomized controlled trial

**DOI:** 10.1186/1745-6215-12-29

**Published:** 2011-02-02

**Authors:** Nieke A Elbers, Arno J Akkermans, Pim Cuijpers, David J Bruinvels

**Affiliations:** 1 Department of Law, VU University, Amsterdam, The Netherlands; 2 Department of Clinical Psychology, VU University, Amsterdam, The Netherlands; 3 EMGO Institute, VU University Medical Center, Amsterdam, The Netherlands; 4 Amsterdam Interdisciplinary Center of Health and Law, The Netherlands; 5 Dutch Society of Occupational Medicine (NVAB), Utrecht, The Netherlands; 6 Coronel Institute of Occupational Health, Academic Medical Center, Amsterdam, The Netherlands

## Abstract

**Background:**

Research has shown that current claims settlement process can have a negative impact on psychological and physical recovery of personal injury (PI) victims. One of the explanations for the negative impact on health is that the claims settlement process is a stressful experience and victims suffer from renewed victimization caused by the claims settlement process. PI victims can experience a lack of information, lack of involvement, lack of 'voice', and poor communication. We present the first study that aims to empower PI victims with respect to the negative impact of the claims settlement process by means of an internet intervention.

**Methods/design:**

The study is a two armed, randomized controlled trial (RCT), in which 170 PI victims are randomized to either the intervention or control group. The intervention group will get access to a website providing 1) an information module, so participants learn what is happening and what to expect during the claims settlement process, and 2) an e-coach module, so participants learn to cope with problems they experience during the claims settlement process. The control group will get access to a website with hyperlinks to commonly available information only. Participants will be recruited via a PI claims settlement office. Participants are included if they have been involved in a traffic accident which happened less than two years ago, and are at least 18 years old.

The main study parameter is the increase of empowerment within the intervention group compared to the control group. Empowerment will be measured by the mastery scale and a self-efficacy scale. The secondary outcomes are perceived justice, burden, well being, work ability, knowledge, amount of damages, and lawyer-client communication. Data are collected at baseline (T0 measurement before randomization), at three months, six months, and twelve months after baseline. Analyses will be conducted according to the intention-to-treat principle.

**Discussion:**

This study evaluates the effectiveness of an internet intervention aimed at empowerment of PI victims. The results will give more insight into the impact of compensation proceedings on health over time, and they can have important consequences for legal claims settlement. Strengths and limitations of this study are discussed.

**Trial registration:**

Netherlands Trial Register NTR2360

## Background

In the Netherlands, each year about 50.000 people file a PI liability claim. Research has shown that the current claims settlement process has a negative impact on personal injury (PI) victims' health and well-being [1]. Most of the studies that investigated the influence of litigation or compensation on health show that PI victims who are involved in litigation are less likely to return to work [2], have more disability, worse health outcomes [3,4], increased pain intensity and decreased physical functioning [5-8], and more symptoms of depression, anxiety and distress [9-14] than non-litigating PI victims.

The negative impact of compensation proceedings on health is often explained by the theory that being involved in claims settlement creates an unconscious incentive for victims not to get better as long as the settlement lasts, which is called *secondary gain *[15]. However, the negative impact of compensation proceedings on health can also be explained by the fact that the claims settlement process is a stressful experience and victims suffer from renewed victimization caused by the claims settlement process, which is called *secondary victimization *[16]. Claims settlement focuses solely on the assessment of monetary damage, whereas victims' immaterial needs are often neglected. Victims can experience a lack of information, lack of involvement, and lack of opportunity to tell their site of the story ('voice'), they can get the feeling they are being mistrusted and not taken seriously, and the communication can be poor [17,18].

The importance of providing information, an opportunity for 'voice' and a respectful treatment, is supported by the theory of *procedural justice *[19], arguing that the perception of justice is more determined by procedural aspects and the way a decision is reached, rather than the outcome itself. A lack of procedural justice was found to be related to negative emotions such as anger, frustration, anxiety [20], stress and depression [21], whereas procedural fairness in the sense of getting the opportunity to voice their opinion was found to be a stress reducing factor [22].

Considering the fact that a compensation proceeding has a negative impact on health, we expect that there is a need for an intervention tackling the negative aspects of the claims settlement procedure. With respect to providing information, respectful treatment and participation of PI victims, the professionals involved in the settlement process (e.g. loss adjusters, legal representatives on both sides, medical experts, etc.) should of course play an important role. However, in order not to be totally dependent on the quality of the services of these professionals, a self-help intervention in which victims can learn to cope with the negative aspects of the claims settlement process could be a promising alternative approach. There is one study that applied relaxation sessions 'to cope with stressful events (e.g. RTC-related litigation hearings)' (p.544). However, this was only a very small element within a cognitive behavioral treatment for post traumatic stress [23]. A self help intervention which primarily focuses on the claims settlement process has not been developed yet.

In developing an intervention to tackle the negative impact of compensation proceedings, much can be learned from health research, in which many self-help interventions have already been developed for a wide range of health problems, e.g. asthma, eating disorders, weight control, HIV, physical activity, headache, insomnia, cancer, diabetes, post-traumatic stress, depression, anxiety, etc. The methodology of these self-help interventions is also widely differing, but generally, they are designed to improve disease management and provider-patient communication [24,25]. A lot of the self-help interventions contain cognitive behavioral therapy elements, challenging dysfunctional cognitions and behavioral patterns related to the health problem.

Self-help interventions are increasingly offered through the Internet ('e-health'). Providing self-help interventions via the Internet has several advantages over usual care: it is anonymous, it has low costs, it can be accessed at any time, at any place, it takes no travel time and there is no waiting list. Internet interventions were found to increase patient empowerment, i.e. (disease specific) self-efficacy and mastery [25], improve knowledge and behavioral outcomes [24], reduce health problems, e.g. pain and headache [26], and reduce depression and anxiety [27].

Considering the fact that self-help internet interventions are found to be effective in improving health in a wide range of health problems, we expect that self-help internet interventions can very well be applied to PI victims.

In this article, we present the first study that aims to empower PI victims with respect to the negative aspects of the claims settlement process by means of an internet intervention, providing 1) an information module, so PI victims learn what is happening and what to expect during the claims settlement process, and 2) an e-coach module, a course with cognitive-behavioral techniques, so PI victims can learn to cope with the negative aspects of the claims settlement process. In developing the intervention, we extrapolated the existing e-health knowledge to the legal domain. The results of this study will give more insight into the impact of compensation proceedings on health over time, and can have important consequences for legal claims settlement and the provision of legal services to individual citizens in general, as is further elaborated in the discussion.

## Methods

### Study design

The study is a two armed, randomized controlled trial (RCT). Participants are randomized to either the intervention group or control group. The study protocol has been reviewed by the Medical Ethics Committee of the VU University Medical Center (registration number 2010/123).

### Study population

In the Netherlands, each year about 50.000 PI victims file a liability claim. Participants (n = 170) will be recruited through claims settlement office Korevaar Van Dijk http://www.korevaarvandijk.nl. Korevaar Van Dijk is situated in the Randstad (i.e. urban agglomeration of Western Holland). Korevaar Van Dijk represents about 800 new clients each year. About 95% of the clients are traffic accident victims. 40% have whiplash injuries.

### Inclusion criteria

Inclusion criteria are:

- Being a road traffic victim

- Accident happened less than two years ago

- Having access to the internet and an email address

- Being at least 18 years old

- Being fluent in Dutch language

### Sample size

The primary outcome variable of this study is empowerment, which is measured by the Mastery Scale [28]. This scale has a range of 7 to 35. To be able to show a medium effect size (Cohen's d of 0.50) using a power of 80% and a two-sided alpha of 5%, we will need 63 participants per group. Taking into account a loss to follow-up of 25%, we will need to randomize 85 participants per group. Having two groups (intervention group and control group), a total of 170 participants is needed.

### Randomization

After baseline measurement, participants are randomized by an independent researcher to either the intervention or the control group. Stratified randomization will insure that new cases (accident happened 0-1 year ago) and older cases (accident happened 1-2 years ago) will be equally divided over the intervention and control condition. The allocation schedule will be made by a computerized random number generator that will generate fixed blocks of 20. Participants and researcher will be blind for allocation.

### Intervention

The intervention is an interactive website http://www.gripopmijnzaak.nl, providing 1) general claims settlement information, so participants learn what is happening and to expect during the claims settlement process, and 2) e-coach support, so that participants can learn to cope with worries and problems, and 3) frequently asked questions with answers. See additional file 1 for a print screen of the website.

#### Information

The information module consists of five subheadings: claims settlement process, representative, opposite party, social services, and conflict resolution. In the first subheading, we show participants that the claims settlement process can be divided in four phases: 1) assessment of liability, 2) medical assessment, 3) assessment of earning capacity & rehabilitation, and 4) assessment of damages. Within each phase, we discuss: a) the important concepts, e.g. what is 'liability', what is 'contributory negligence', b) the steps, e.g. first the accident information is collected, then liability is established, c) the turnaround time, e.g. liability should be established within three months, and d) the possible bottlenecks, e.g. the opposite party denies liability, or claims that the claimant is guilty of contributory negligence. Because the claims settlement process is divided in phases, participants are able to keep up what is happening during claims settlement, and what will happen in the future.

Second, we discuss the legal professionals representing PI victims. In the Netherlands, over 95% of PI claims are settled out of court. In the negotiations with the liable party, victims can be represented by three kinds of legal professionals: lawyers who are members of the bar, often also specialized in PI claims (working at a law firm), legal representatives who are not working at the bar (working at a specialized PI claims settlement office), and lawyers working for a legal expenses insurance company. These three different kinds of legal professionals are introduced and the differences are explained. Furthermore, the applicable guidelines and codes of conducts are introduced and discussed, so that participants learn what they can expect from their lawyer. We discuss the costs of legal aid and the different remuneration arrangements that are commonly made in the Netherlands, and we discuss the options in case participants are unsatisfied with their lawyer.

Third, we provide information about the opposite party. In the Netherlands, compensation for traffic accident victims is ruled by general tort law. We show that there are three different kinds of opposite parties: normally, the opposite party is a private insurance company, sometimes a traffic accident guarantee fund, and even more rarely a road maintenance authority. We also describe the codes of conduct for insurance companies, so that participants learn what to expect from the opposite party.

The fourth information section deals with social services that are relevant for people with disability. Here, we discuss the statutory benefits that PI victims can be eligible for, such as help and support in housekeeping and care, and social security benefits.

Fifth, we explain three different options for resolution of conflicts that may arise during the claims settlement process. Participants are informed that personal contact with the opposite party is a first step to prevent a rising conflict. If personal contact does not prevent or solve the conflict, some conflicts are suitable for mediation. The final option is to go to court. Here, we included information about the different court procedures, the costs involved, and the time a court procedure takes.

#### E-coach

The e-coach module consists of the Dutch internet-based problem solving intervention by Van Straten et al [29], that is based on the self examination therapy by Bowman et al [30]. This problem solving intervention is an online course of five weekly lessons, in which patients identify their problems and learn how to cope with them. Participants learn to 1) determine worries and problems, 2) tackle solvable problems in six steps, 3) think less negatively about unimportant problems, 4) accept unsolvable problems, and 5) make a future plan. Each lesson consists of reading, examples and assignments. The intervention was found to be effective in reducing depression, anxiety and work related stress [29,31].

We applied the problem solving intervention to the problems experienced by PI victims and hence focuses on the burdening aspects of the claims settlement process, and problems coping with the accident and/or the injury. The problems and examples in the course are rewritten into problems and examples that are recognizable for PI victims, and some relevant cognitive behavioral techniques are added. In lesson 2, we added communication techniques, i.e. to express thoughts in an objective en non-accusing way. In lesson 3, we added a paragraph about thinking errors (e.g. drawing wrong conclusions). In lesson 4, we turned the examples of unsolvable problems into dealing with (permanent) disability and into coping with certain unpleasant but unsolvable aspects of the claims settlement process, such as the plaintiff's obligation to prove the injury and the damage, and the defendants' right to contradict the evidence.

We developed three different examples of PI victims, all suffering different problems during claims settlement. Our first example is Mark, a 25 year-old construction worker who suffers back and hip injury. His problem with claim settlement concerns the disagreement about the compensation (fourth phase, assessment of damages). Furthermore, he has difficulties coping with the injury.

Our second example is Susan, a 41 year-old secretary, who has whiplash injury. The problem she experiences during the claims settlement process concerns the medical assessment of her whiplash injury (second phase, medical assessment). Her other problem is her financial insecurity.

The third example is Philip, a 53 year-old IT worker, who has a broken leg. His problem with the claims settlement process concerns the fact that the insurance company claims that he is guilty of contributory negligence (first phase, assessment of liability). The other problem is that he is hindered by accident trauma.

Participants are given feedback by email on homework assignments they make. In principle, the feedback is given by a psychologist (i.e. the primary investigator of this study). If the work load turns out to be too high, Victim Support Netherlands will be contacted for help.

#### Frequently asked questions

The website also contains a 'frequently asked questions' section, in which ten frequently asked questions are answered. For example: 'Why does the settlement of my claim take so long?', 'How much compensation will I get?'. Most answers can also be found in the information module.

#### Control group

The control group will get access to the sham website, containing hyperlinks to already existing websites with 1) claims settlement information, the Dutch Judiciary, and the Dutch social security organization, and 2) non-profit support organizations, and companion groups.

#### Focus group

After we developed the intervention, we held a focus group in which six PI lawyers ('plaintiffs') and five representatives of insurance companies ('defendants') were present. The participants of the focus group expected that the intervention will meet the needs of PI victims and will improve client lawyer relationship and hence involvement of the client. Furthermore, the used language was found to be comprehensible, simple, clear and neutral. With respect to the information module, some textual changes were made to make the information more accurate and neutral. We included their suggestions for frequently asked questions and we included their tips for PI victims in case the opposite party will visit them at home. We removed the hyperlinks to two television programs, in which PI victims were interviewed about their bad experiences with either their lawyers or with the opposite party, because these cases are exceptions and could feed 'polarization'. Finally, based on the advice of the focus group, we decided not to add whiplash as a separate topic, but to discuss whiplash as a 'bottleneck', to explain why whiplash injury is more difficult than for example orthopedic injury, and to report whiplash recovery statistics.

#### Pilot

After we incorporated the input from the focus group in the website, we recruited eight PI victims to pilot test the intervention website. These pilot victims were recruited via PI claims settlement office called Hofmans Associates http://www.hofmanshelpt.nl, situated in Amsterdam. We asked the pilot participants to grade the different components of the website, to make suggestions for improvement, to grade the lay-out and language, and to indicate whether they would use the different components of the website themselves. The website was graded well. With respect to the information module, one participant noted that 'there was almost too much information', and one participant commented that the website should include 'more information about bad lawyers'. We decided not to add information about bad lawyers, because we had just removed that kind of information on the advice of the focus group (to avoid 'polarization'). Considering the e-coach module, one participant wrote that 'problems are not always solvable or unsolvable. The question whether the injury will heal completely is not solvable: one can only wait for the outcome'. Although the e-coach already discusses 'learn to live with injury' as an unsolvable problem, we decided to add 'waiting for the injury to heal' to the list of unsolvable problems.

Language and lay-out were graded well. One participant made a final comment that the menu structure, menu readability, and hyperlink system were not very clear, whereas one participant said the contrary: that the website 'is very clear, well organized and plain. Also the references to extra information are very clear', so we decided not to change the lay-out, except from adding a symbol to differentiate between hyperlinks referring to external websites and hyperlinks within our website. All respondents indicated that they would use the information module and the frequently asked questions. Three out of eight respondents said they would use the e-coach module. All participants were send a 10 euro gift voucher incentive.

### Procedure

PI victims will be recruited via the PI claims settlement office Korevaar Van Dijk. All clients will be sent an information leaflet by email, or if no email address is registered, the leaflet will be sent by post. Clients that meet the inclusion criteria and are interested to participate in the study, will be directed to the website http://www.gripopmijnzaak.nl. The website will provide a registration form, where participants will fill in name and email address, inclusion criteria are checked, and informed consent is obtained. After successful enrolment, clients will receive an email with a link to the baseline questionnaire (T0). After the baseline questionnaire is filled in, participants will be randomized by an independent researcher to either the intervention group or the control group. Randomization will be stratified by new cases (accident happened 0-1 year ago) and older cases (accident happened 1-2 years ago).

The intervention group will receive an email with username and password to access the intervention website, the control group will receive an email with username and password to access the control (sham) website. Measurements will take place three months after baseline (T1), six months after baseline (T2) and twelve months after baseline (T3). All measurements are online questionnaires, provided by NetQuestionnaire http://www.netq.nl. Participants will automatically receive an email with a personal link to the questionnaire. Participants who complete all four questionnaires will receive a 20 euro gift voucher.

**Figure 1:**
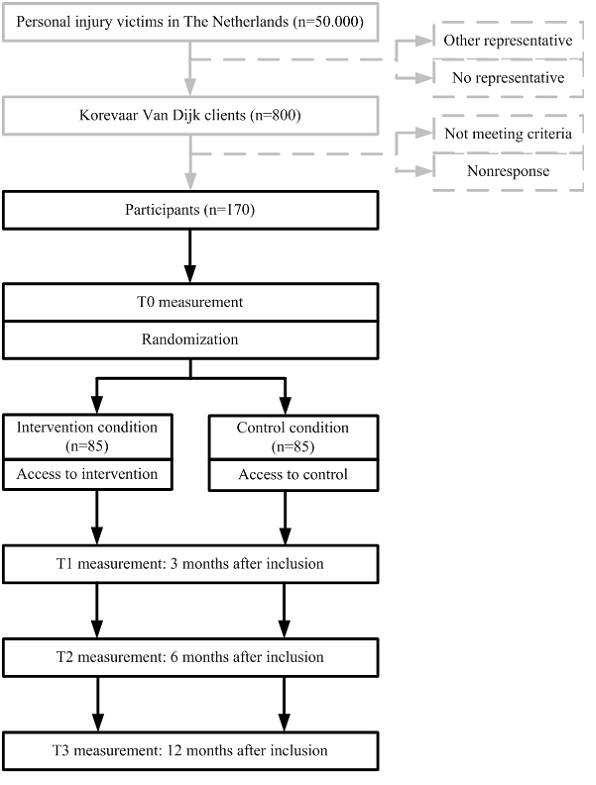
**Study procedure**.

The study design is presented in Figure 1.

### Primary outcome measure

Empowerment will be measured by 1) the Dutch version of the mastery scale [28] and 2) a self-efficacy scale.

#### Mastery

The mastery scale consists of seven items regarding to what extent one experiences control in life. Items are rated on a five point scale with higher scores indicating greater perceived control. The mastery scale has good psychometric properties [28].

#### Self-efficacy

Samoocha and colleagues [25] found that web-based interventions had a significant effect on self-efficacy measured by disease-specific self-efficacy scales, while no effect was found when self-efficacy was measured by general self-efficacy scales. Hence, we developed a specific self-efficacy scale that addresses the three main problem areas that PI victims can face: 1) the claims settlement process, 2) the injury, and 3) the accident. For each problem area, it is questioned whether one is capable i) to tackle solvable problems, ii) not to worry about irrelevant problems and iii) accept unsolvable problems (i, ii and iii are the main skills that are addresses by the e-coach module). The questionnaire consists of nine items and the response scale runs from 0 (cannot do at all) to 10 (highly certain can do). The self-efficacy scale is developed according to the guidelines for the development and construction of self-efficacy scales [32].

We will conclude an enhancement in empowerment if both scales show a positive effect, or if one of the two scales (mastery or self-efficacy) shows a positive effect and the other scale does not show a negative effect.

### Secondary outcome measure

#### Perceived justice

Perceived justice will be measured by the organizational justice questionnaire developed and validated by Colquitt [33]. This questionnaire consists of four subscales: procedural justice (seven items with respect to the 'procedures to come to your compensation'), distributive justice (four items with respect to 'your compensation'; this subscale is only questioned when the participant has indicated that the claim is settled), interactional justice (four items concerning 'your lawyer'), and informational justice (five items concerning 'your lawyer'). In total, twenty items will be questioned with five option answer categories (1 = not at all, 5 = always). We applied a Dutch translation by Van Prooijen [34] of the procedural- (α = 0.74), distributive-, and interactional justice scale (not reported in the article) to our target population. We did not find a Dutch translation of the informational justice scale, so we translated the informational justice scale in line with the other scales. Additionally, the interactional justice subscale is applied to 'the opposite party'.

#### Burden

Participants will indicate to what extent they considered the claims settlement process to be a burden on a ten point scale (1 = not at all, 10 = very much).

#### Well-being

Well-being will be measured by 1) three subscales of the SCL-90 [35] (i.e. depression, anxiety, and somatization: 38 items), with a five point answer scale (1 = not at all, 5 = very much), and 2) the EQ-5D [36], which is a validated tool for measuring quality of life. It consists of i) five items (mobility, self-care, usual activities, pain/discomfort, and anxiety/depression) with a three point answer scale (no problems, some problems, or extreme problems) and ii) a visual analogue scale questioning the respondent's self-rated health (0 = worst imaginable health state, 100 = best imaginable health state).

#### Work ability

Work ability will be administered by the first three items of the Dutch version of the Work Ability Index [37], determining individual work capacity. Work is defined as a paid job, but also studies, housekeeping, care for fellow human beings, and volunteer aid. The first question asks subjects to rate their current work ability compared to their lifetime best on an eleven point scale (0 = completely unable to work, 10 = work ability at it best). The second and third question ask participants to judge their current work ability considering respectively the physical - and the mental demands of their work (1 = very bad, 5 = very good).

#### Knowledge of claims settlement

Knowledge of claims settlement will be measured by a self developed questionnaire with six items, covering the different components of the information module of the intervention. Participants are asked to what extent they know: 1) the state of affairs regarding the settlement of their claim, 2) what to expect of the claims settlement procedure, 3) what to expect from their lawyer, 4) what to expect from the opposite party, 5) which social services to count on, and 6) what to do in case of conflict. The questionnaire has a five point answer scale (1 = not at all, 5 = a lot).

#### Compensation

Participants will be asked to estimate the amount of compensation they expect to receive. In case the claim is settled, they are asked to fill in the amount of compensation they have received.

#### R-C Communication

The lawyer will be asked to rate the communication with the client (participant) on a scale from 1 to l0.

### Other variables

#### Demographic variables

Demographic variables are: 1) gender, 2) birth date, 3) place of residence, 4) country of birth, 5) educational level (five answer options), and 6) whether the respondent had a paid job at the time of the accident (employer, self-employed, or unemployed).

#### Accident

Questions concerning the accident are: 1) participant's means of transport when the accident happened (motorized or not motorized), 2) date of accident, and 3) the extent in which the offender can be blamed for the accident (1 = not at all, 5 = very much).

#### Injury

Injury details will be measured by questioning: 1) what body part is injured (a) shoulder, arm or hand, b) head or neck, c) hip, leg or foot, d) trunk or back; multiple answers possible), 2) whether one was admitted to the hospital (if yes, how many days), and 3) whether the injury can be objectified (e.g. by scan).

#### Claims settlement

Claims settlement details are: 1) date of first contact with lawyer, 2) name of lawyer, and 3) name of opposite party.

#### Website satisfaction

Website satisfaction will be measured by one question asking to rate the website on a scale from 1 to10.

#### Website usage

Website usage is the amount of webpage views, which is automatically registered in the *back office *of the website.

An overview of measurements is displayed in table 1.

**Table 1:** Schedule of measurements

Measurement		T0	T1	T2	T3
		Baseline	3 months	6 months	12 months
Empowerment	Mastery scale	7	7	7	7

Self efficacy	Self developed items	9	9	9	9

Justice	Organizational justice	20	20	20	20
	
	Self developed items	4	4	4	4

Burden	Self developed item	1	1	1	1

Well being	SCL-90 (3 subscales)	38	38	38	38
	
	EQ-5D	6	6	6	6

Work ability	Work ability index	3	3	3	3

Knowledge	Self developed items	6	6	6	6

Compensation claim	Self developed item	-	-	-	1

R-C communication	Self developed item	-	-	-	1

Demographics	Self developed items	6	-	-	-

Accident	Self developed items	3	-	-	-

Injury	Self developed items	3	-	-	-

Claims settlement	Self developed items	3	-	-	-

Website satisfaction	Self developed item	-	-	-	1

Website usage		Number of webpage views			

**Total number of questions**		**109**	**94**	**94**	**97**

### Statistical analysis

Descriptive statistics (categorical and continuous variables) will be analyzed by respectively chi-square and t-test. All analyses will be conducted according to the intention-to-treat principle. Missing values will be imputed with regression imputation techniques. Differences between the intervention group and control group will be evaluated by two tailed tests at significance level of 5% (p < 0.05). Short term (T1, T2) and long term (T3) effects will be analyzed by a repeated measure analysis. Finally, the results of the intention-to-treat analyses will be compared to the results of the per-protocol analyses.

## Discussion

This study is the first to empower PI victims with respect to the negative aspects of the claims settlement process by means of a internet intervention. Below, we will discuss the strengths and limitations of this study.

From a scientific point of view, the results will give more insight into the impact of compensation proceedings on health over time and the phenomena of secondary gain and secondary victimization. Because this study is the first internet intervention applied to legal practice, the study will provide interesting data whether a self-help intervention is applicable to our target population. Furthermore, the website usage data will reveal what kind of PI victims will use which modules and how often.

If we succeed in improving health of PI victims, the results of this study can have important consequences for legal claims settlement. If this research shows that empowerment via an interactive website has a positive influence on the well-being and health of PI victims, than our website has a clear potential to become standard service in legal practice, and possibly even an obligatory service to PI victims, considering the fundamental rule in law that recovery has priority over monetary compensation (*restitutio in integrum*). A positive outcome would constitute the empirical basis for the development of legal rules that would make legal professionals to adhere a more victim-friendly and recovery oriented way of settling PI claims. A further step could be the development of comparable websites, designed to empower individual citizens who are entangled in comparable burdensome legal procedures, e.g. in the field of labor law, housing law, consumer law, administrative law, and civil law in general. Furthermore, the intervention and the results of the study will also be interesting for victimology and criminology studies.

Offering a self-help intervention by which PI victims can keep up with what is going on during the claims settlement process and by which they can learn to cope with problems and worries, could be a promising alternative approach for a problem that until now is only being encountered by educating the legal professionals on more client friendly claims settlement processes.

Another strength of this study is that the intervention is offered through the internet. Hence, the intervention can be accessed easily, at home, and at any time, which is especially advantageous for our target population that is disabled and often immobile. Furthermore, because of the internet, we are able to reach a large audience at low costs, and anonymously, which is beneficial considering the fact that we are providing a service for a hardly acknowledged health problem.

The fact that we choose to recruit participants via only one claims settlement office has both advantages and disadvantages. The first advantage of recruitment via a claims settlement office (compared to indirect recruitment via the media) is that we assume to have a relative smooth inclusion of participants, because we can directly approach a large number of PI victims. Second, we assume that lawyers working in the same claims settlement office have a similar method of claims settlement, so that 'method of claims settlement variability' will not be a confounder. However, recruitment via only one office also implies two possible selection biases. First, it might be that the characteristics of clients of this particular claims settlement office may differ from clients of other offices. Second, this claims settlement office is one of the first offices in the Netherlands which offers their clients online access to their claims settlement dossier, a service which will be a standard service in the future, but at the moment is not usual care.

A limitation of the study is that the claims settlement process will be different for all participants: different length, different steps, different pace, and different problems. Because of this variability, we cannot investigate whether there are moderating factors influencing the study outcomes. Furthermore, some of the claims will be settled before the end of the study. Participants whose claim is already settled early in the study, will use the intervention for a short time only and their reports will be influenced by the perceived fairness of the compensation they received (distributive justice), so they need to be analyzed differently. However, it is unclear whether the number of settled claims will be large enough to draw conclusions about this subgroup.

A second limitation concerns the generalization of the study results. Because our participants are traffic accident victims, further research is needed to find out whether the results can be generalized to other kinds of PI victims, such as victims of medical malpractice, workplace accidents, and violent assaults. Furthermore, because of international variety in compensation proceedings and legal services delivery [38], we should be careful to generalize the study results to countries with different ways of claims settlement processes. General tort law might give different needs and experiences than a no-fault system. The same goes for claims that are settled out of court versus claim proceedings in court, or PI victims who are represented by a lawyer compared to PI victims who are not represented by a lawyer.

The results of this study are expected in early 2012.

## Competing interests

The authors declare that they have no competing interests.

## Authors' contributions

AA obtained funding for the study. All authors contributed to the design of this study. NE and AA developed the information module. NE adapted the e-coach module for the target population. NE wrote the first version of the manuscript. All authors contributed to next versions of the manuscript. All authors have read and approved the final version of the manuscript.

## Supplementary Material

Additional file 1 **Print screen **http://www.gripopmijnzaak.nl. Print screen of the intervention website http://www.gripopmijnzaak.nl. Click here for file
