# Supplementary figures and images for: Empowerment of personal injury victims through the internet: design of a randomized controlled trial
Source: Trials. 2011 Feb 2;12:29. doi: 10.1186/1745-6215-12-29 (PMC3038943; doi:10.1186/1745-6215-12-29)

# Additional files

### Additional file 1 – Print screen www.gripopmijnzaak.nl

#
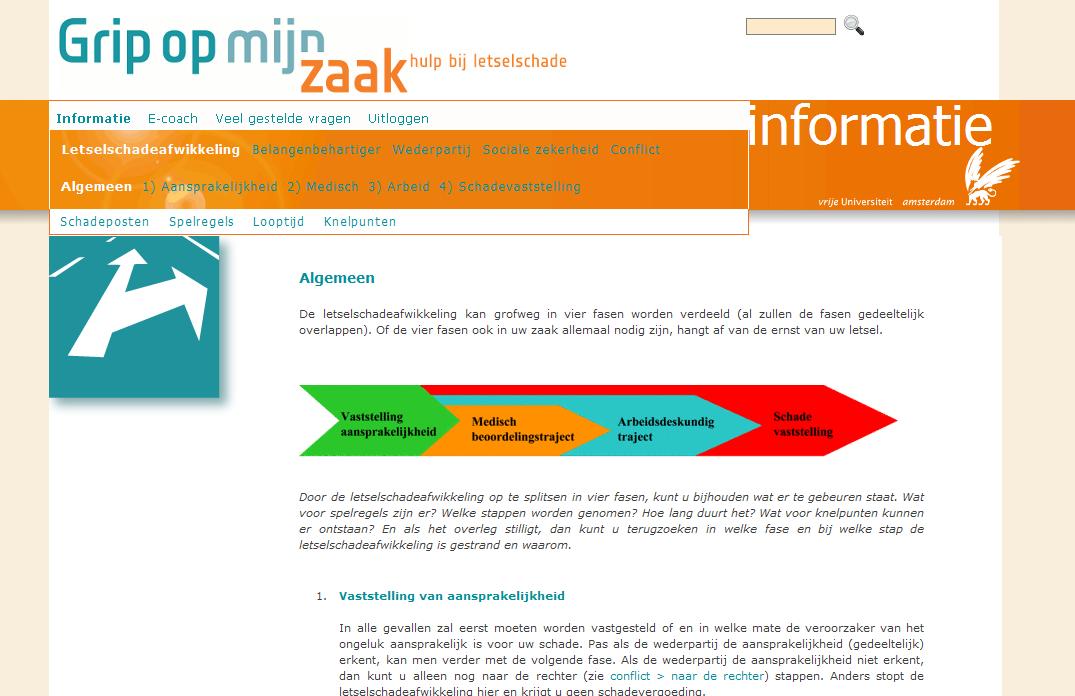

Supplement: Additional file 1 — Print screen http://www.gripopmijnzaak.nl. Print screen of the intervention website http://www.gripopmijnzaak.nl. [file 1745-6215-12-29-S1.DOC]
